# Loss of G9a does not phenocopy the requirement for Prdm12 in the development of the nociceptive neuron lineage

**DOI:** 10.1186/s13064-023-00179-7

**Published:** 2024-01-02

**Authors:** Panagiotis Tsimpos, Simon Desiderio, Pauline Cabochette, Philippe Poelvoorde, Sadia Kricha, Luc Vanhamme, Coralie Poulard, Eric J. Bellefroid

**Affiliations:** 1 ULB Neuroscience Institute (UNI), Université Libre de Bruxelles (ULB), Gosselies, B- 6041 Belgium; 2 Department of Molecular Biology, Institute of Biology and Molecular Medicine, IBMM, Université Libre de Bruxelles, Bruxelles, Belgium; 3 Cancer Research Cancer of Lyon, Université de Lyon, Lyon, F-69000 France; 4 Inserm U1052, Centre de Recherche en Cancérologie de Lyon, Lyon, F-69000 France; 5 CNRS UMR5286, Centre de Recherche en Cancérologie de Lyon, Lyon, F-69000 France

**Keywords:** Dorsal root ganglia, Neurogenesis, Somatosensory neurons, Nociceptors, G9a, Prdm12

## Abstract

Prdm12 is an epigenetic regulator expressed in developing and mature nociceptive neurons, playing a key role in their specification during neurogenesis and modulating pain sensation at adulthood. In vitro studies suggested that Prdm12 recruits the methyltransferase G9a through its zinc finger domains to regulate target gene expression, but how Prdm12 interacts with G9a and whether G9a plays a role in Prdm12’s functional properties in sensory ganglia remain unknown. Here we report that Prdm12-G9a interaction is likely direct and that it involves the SET domain of G9a. We show that both proteins are largely co-expressed in dorsal root ganglia during early murine development, opening the possibility that G9a plays a role in DRG and may act as a mediator of Prdm12’s function in the development of nociceptive sensory neurons. To test this hypothesis, we conditionally inactivated G9a in neural crest using a Wnt1-Cre transgenic mouse line. We found that the specific loss of G9a in the neural crest lineage does not lead to dorsal root ganglia hypoplasia due to the loss of somatic nociceptive neurons nor to the ectopic expression of the visceral determinant Phox2b as observed upon *Prdm12* ablation. These findings suggest that Prdm12 function in the initiation of the nociceptive lineage does not critically involves its interaction with G9a.

**Supplementary Information:**

The online version contains supplementary material available at 10.1186/s13064-023-00179-7.

## Introduction

 The bodily ability of vertebrates to discriminate and respond to a wide array of salient stimuli resides in the great diversity of neuron subtypes whose cell bodies are found in dorsal root ganglia (DRG) and build a discriminative sensory relay between the periphery and the central nervous system. This neuronal diversity arises during development when specific transcriptional programs are initiated that bias the fate of neural crest-derived somatosensory progenitors into one of the three cardinal somatosensory lineages. These three main subtypes of somatosensory neurons can be discriminated early in development based on the selective expression of tyrosine kinase neurotrophic receptors [1–4]. Tyrosine kinase receptor A (TrkA)-is expressed in developing lightly or unmyelinated nociceptive neurons of small or medium diameter which mostly respond to noxious stimuli but are also involved in temperature or itch sensing [5] and in unmyelinated low-threshold mechanoreceptive (LTMR) neurons involved in pleasurable touch [6–8]. Ret, TrkB and TrkC are expressed in more myelinated LTMR neurons that convey innocuous touch sensation and proprioception. These early developmental selective expressions eventually evolve over time as sensory neurons mature and diversify into more specialized subtypes, with the wider diversification seemingly arising in the TrkA lineage [3, 9].

Over the last decades, a comprehensive understanding of the main transcriptional regulators guiding the early development and diversification of somatosensory neurons has been acquired. Notably, molecular players required for the emergence and diversification of the TrkA lineage have been identified [3, 4]. Among them, the transcriptional regulator Prdm12 stands at the root of the specification of this lineage. Indeed, Prdm12 function is critical for the emergence of the entire pool of neurons arising from immature TrkA-expressing sensory neuron precursors as well as for TrkA expression itself [10–14]. Accordingly, in human, patients harbouring homozygous mutations of *PRDM12* suffer from congenital insensitivity to pain (CIP), a rare developmental disorder associated with depletion of somatosensory fibers allowing the detection of noxious stimuli [15, 16]. Moreover, as *Prdm12* remains expressed in mature nociceptive neurons and its induced conditional ablation at adulthood alters some pain-related behaviours, it has been hypothesized as a potential new therapeutic target to treat pain related diseases [16, 17]. However, to approach the therapeutic potential of Prdm12, a deeper understanding of its mode of action needs to be first established.

Prdm12 is a member of the PRDM family of epigenetic (ZF) zinc-finger transcriptional regulators playing roles in many developmental processes and diseases [18–21]. While some PRDM family members possess intrinsic histone methyltransferase activity through their SET related PR domain, it appears not to be the case for Prdm12 which has been shown to be able to form a complex when overexpressed in HEK293T cells with G9a, a histone methyltransferase (HMT) that dimethylates histone H3 at lysine 9 (H3K9me2) in euchromatin, a hallmark of epigenetic repression [22]. While some evidence have been obtained in *Xenopus* suggesting that this interaction with G9a may be functionally relevant for Prdm12’s activity during spinal cord neurogenesis and in preplacodal ectoderm [23, 24], no such in vivo evidence is available in the developing nociceptive lineage.

In this study, using co-immunoprecipitation assays in HEK293T cells, we show that Prdm12 interacts with the SET domain of G9a. To evaluate G9a function in sensory neurogenesis and possible importance for Prdm12 functions in developing DRG, we generated *G9a* conditional knockout murine embryos (*G9a* cKO) in which *G9a* is selectively invalidated in the neural crest lineage. We report here that the loss of G9a does not phenocopy the requirement of Prdm12 for the initiation of the nociceptive lineage. These data suggest that G9a is not instrumental for Prdm12 function during somatosensory neurogenesis.

## Results

### Prdm12 interacts via its zinc fingers with the SET domain of G9a

**Fig. 1:**
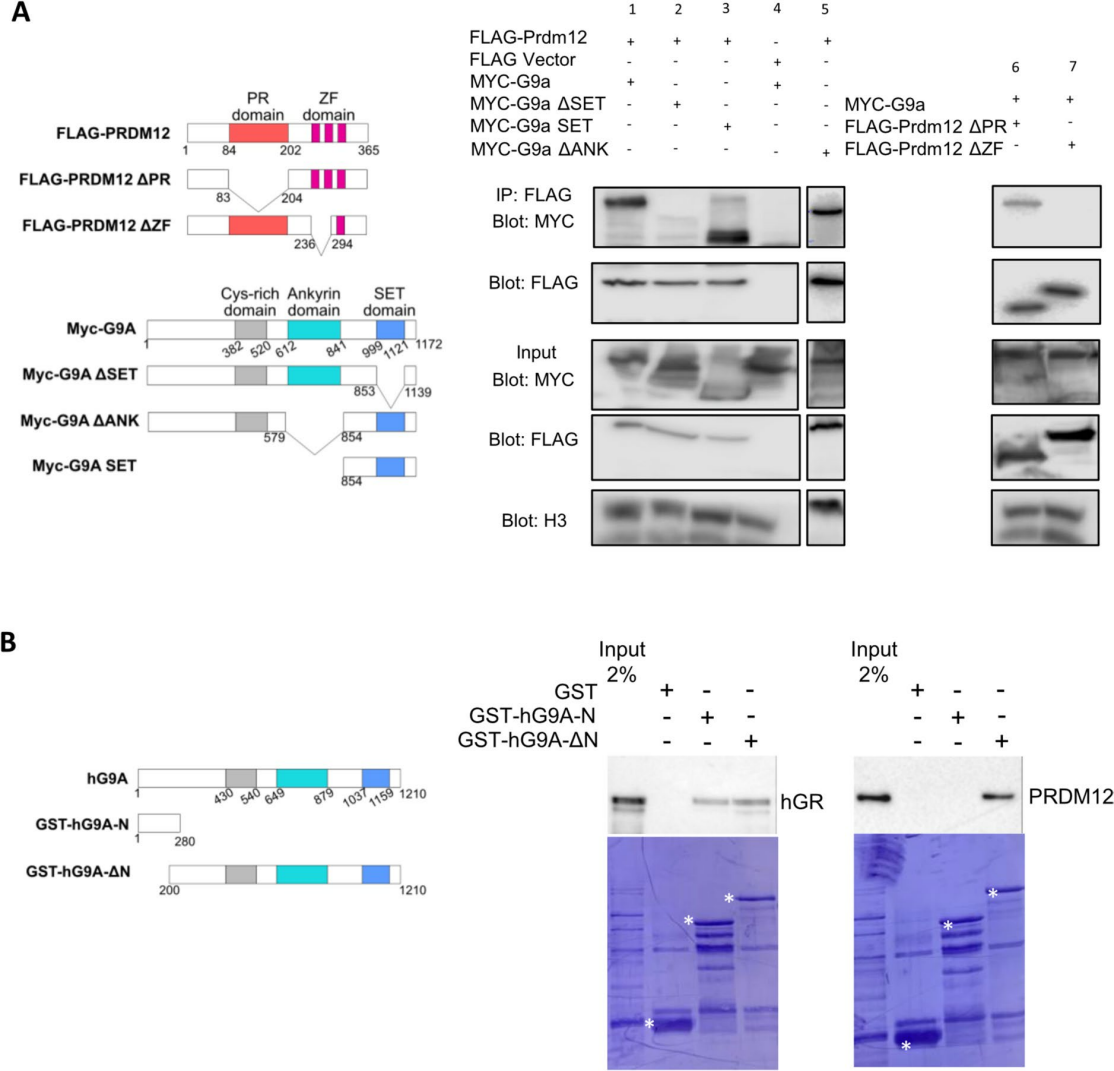
Prdm12 interacts via its zinc fingers with the SET domain of G9a. **A** HEK293T cells were transfected with the indicated plasmids. Schematic diagrams of the Flag-PRDM12 and Myc-G9A WT and deletion mutants used are shown on the left. An empty FLAG plasmid was used as a control. Lysates were immunoprecipitated with anti-Flag antibodies. Immunoprecipitates and 5% of the input were then subjected to western blot analysis with anti-Flag or anti-Myc antibodies. **B** Full-length human Prdm12 or human glucocorticoid receptor (hGR) was synthesized in vitro and incubated with GST or GST fused to hG9a fragments bound to glutathione-agarose beads as indicated. Bound hPrdm12 and hGR proteins were detected by immunoblot with an anti-Flag antibody. A 2% input sample was loaded for comparison. The corresponding Commassie-stained gels are shown

Previous studies have highlighted the ability of Prdm12 to interact with G9a. G9a contains a SET domain that is responsible for its H3K9 methyltransferase activity and mediates homo- or heterodimerization with the related G9a-like protein (GLP) and a Cys-rich ring finger-like domain involved in protein-protein interactions and recruitment of the methyltransferases to specific sites in chromatin [25, 26]. To define which region of G9a is responsible for the interaction with Prdm12, Myc-G9a deletion mutants were generated that eliminate some of the conserved domains of the protein (MYC-G9a ΔSET, ΜYC-G9a ΔΑΝΚ, MYC-G9a SET). Constructs encoding these mutants were co-transfected in HEK293T cells together with a construct encoding a Flag tagged version of mPrdm12. In co-immunoprecipitation (Co-IP) assays, we found that while the MYC-G9a ΔANK and MYC-G9a SET proteins bind to Flag-Prdm12, MYC-G9a ΔSET was unable to do so (Fig. 1A). As reported previously [22], a mutant version of mPrdm12 lacking the ZF domain was unable to interact with G9a. Next, we performed GST pull-down experiments, using two purified GST-G9a fusion proteins, one encoding the N-terminal part of human G9a consisting of amino acids 1-280 and the other containing amino acids 200–1210. In those binding assays, we used full length Flag-human Prdm12 and as a positive control, a Flag human glucocorticoid receptor protein (hGR) as rat hGR binds to the N-terminal part of mG9a (aa 1-330) [27]. Both proteins were produced by in vitro transcription/translation and detected by western blot using anti-Flag antibodies. As shown in Fig. 1B, Flag-hPrdm12 does not interact with purified GST alone or with the fusion encoding the G9a N-terminal fragment but it interacts with the GST-G9a (aa 200–1210) fusion protein. hGR was pulled-down by both GST-G9a fusions. These results suggest that Prdm12 directly interacts via its zinc finger domains with G9a and that the SET domain of G9a is necessary and sufficient for this interaction.

### G9a and Prdm12 are co-expressed during somatosensory neurogenesis

**Fig. 2:**
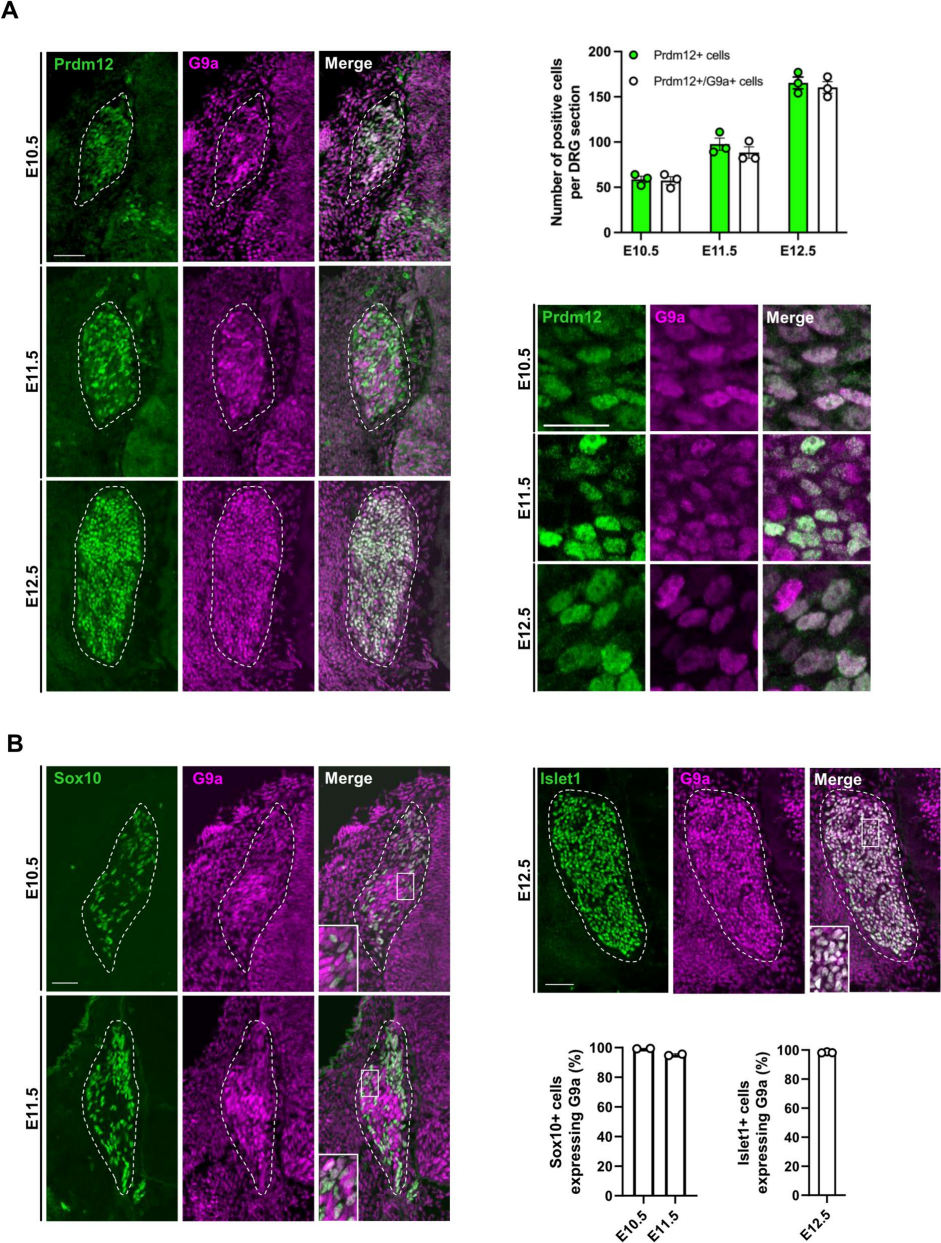
G9a is coexpressed with Prdm12 in developing dorsal root ganglia. **A** Immunostainings for Prdm12 and G9a on coronal sections through DRG of wild-type mouse embryos at indicated stages. DRG are delineated by white dashed lines. High magnification views of representative immunostainings views of representative immunostainings and quantification of the mean number of Prdm12^+^ cells or Prdm12^+^/G9a^+^ cells in coronal sections through DRG of wild-type embryos at indicated stages is shown on the right. Histograms are represented as mean ± SEM. Each dot represents the mean value obtained for an individual biological replicate. Scale bars, 50 µm. **B** Immunostainings for Sox10 and G9a (left panels) and Islet1 and G9a (right panels) on coronal sections through DRG of wild-type mouse embryos at indicated stages. DRG are delineated by white dashed lines. Quantification of the mean number of Sox10^+^or Islet1^+^ cells co-expressing G9a at indicated stages are shown. Histograms are represented as mean ± SEM. Each dot represents the mean value obtained for an individual biological replicate

As a first step to test the hypothesis that Prdm12 functions through G9a to regulate development of the TrkA lineage during somatosensory neurogenesis, we first compared by immunofluorescence the expression of G9a and Prdm12 in DRG of murine embryos from E10.5 to E12.5. Figure 2A shows that G9a is like Prdm12 broadly expressed in DRG at these stages. While all Prdm12^+^ cells colocalized with G9a, some G9a^+^/Prdm12^−^ cells were also clearly visible. To identify which other cell types express G9a, we first compared G9a expression with the pan-sensory neuronal marker Islet1 in DRG of E12.5 embryos and with the neural precursor marker Sox10 in DRG of E10.5 and E11.5 embryos. Results obtained showed that virtually all Islet1^+^ cells and all Sox10^+^ cells in DRG express G9a at these stages (Fig. 2B). At later E15.5 stage, Sox10 whose expression is now restricted to glial cells, appears largely excluded from G9a positive cells (Figure S1). Thus, during early somatosensory neurogenesis, G9a is broadly expressed in neural precursors and differentiating neurons but becomes later excluded from glial precursors. The large co-expression of G9a with Prdm12 in the developing nociceptive neuron lineage suggest that they may functionally interact to control its development.

### Neural crest specific depletion of G9a does not recapitulate the requirement of Prdm12 for genesis of the TrkA-lineage in embryonic DRG

If G9a is a key mediator of Prdm12’s function, then its loss should recapitulate the phenotype observed in *Prdm12* knock-out mice. To test this hypothesis, we generated a *G9a* conditional knockout (cKO) mouse line by crossing *G9a* homozygous floxed mice (exons 26–27) with mice carrying a transgene expressing the Cre-recombinase under the control of the neural crest specific *Wnt1* promoter [28, 29]. The *G9a* cKO mice generated from this strategy survived until late development (E18.5) but never thrived in the mouse litters. Immunofluorescence analysis of G9a expression in E11.5 and E12.5 embryos, when nociceptors are generated, revealed a selective decrease of staining in DRG. Some cells with low level of G9a staining could however still be detected in DRG of G9a cKO suggesting that the G9a loss of function may not be fully penetrant or that there may be Wnt1-independent lineages that develop in the DRG (Figure S2A). By RT-qPCR, using primers taken into the deleted exons (exons 26 and 27) we found a significant reduction of the G9a transcript level in DRG of E14.5 *G9a* cKO compared to WT. This reduction was only of about 50%, may be due to contaminating non DRG tissues expressing G9a collected together with the DRG during the dissection (Figure S2B). H3K9me2, that is catalyzed by G9a was, as expected, strongly reduced in DRG of *G9a* E11.5 cKO compared to controls and this reduction remains detectable at later E14.5 stage. In contrast, H3K9me2 was not lost in *Prdm12* KO embryos, suggesting that Prdm12 does not rely on H3K9me2 marks to mediate its function or that the loss of *Prdm12* alone, which is only one of the putative partners of HMT proteins, is not sufficient to observe a reduction of staining (Figure S2C). Together, these data indicated that *G9a* is selectively inactivated in DRG of *Wnt1-G9a* cKO, which leads as expected to reduced persistent H3K9me2 levels during neurogenesis.

**Fig. 3:**
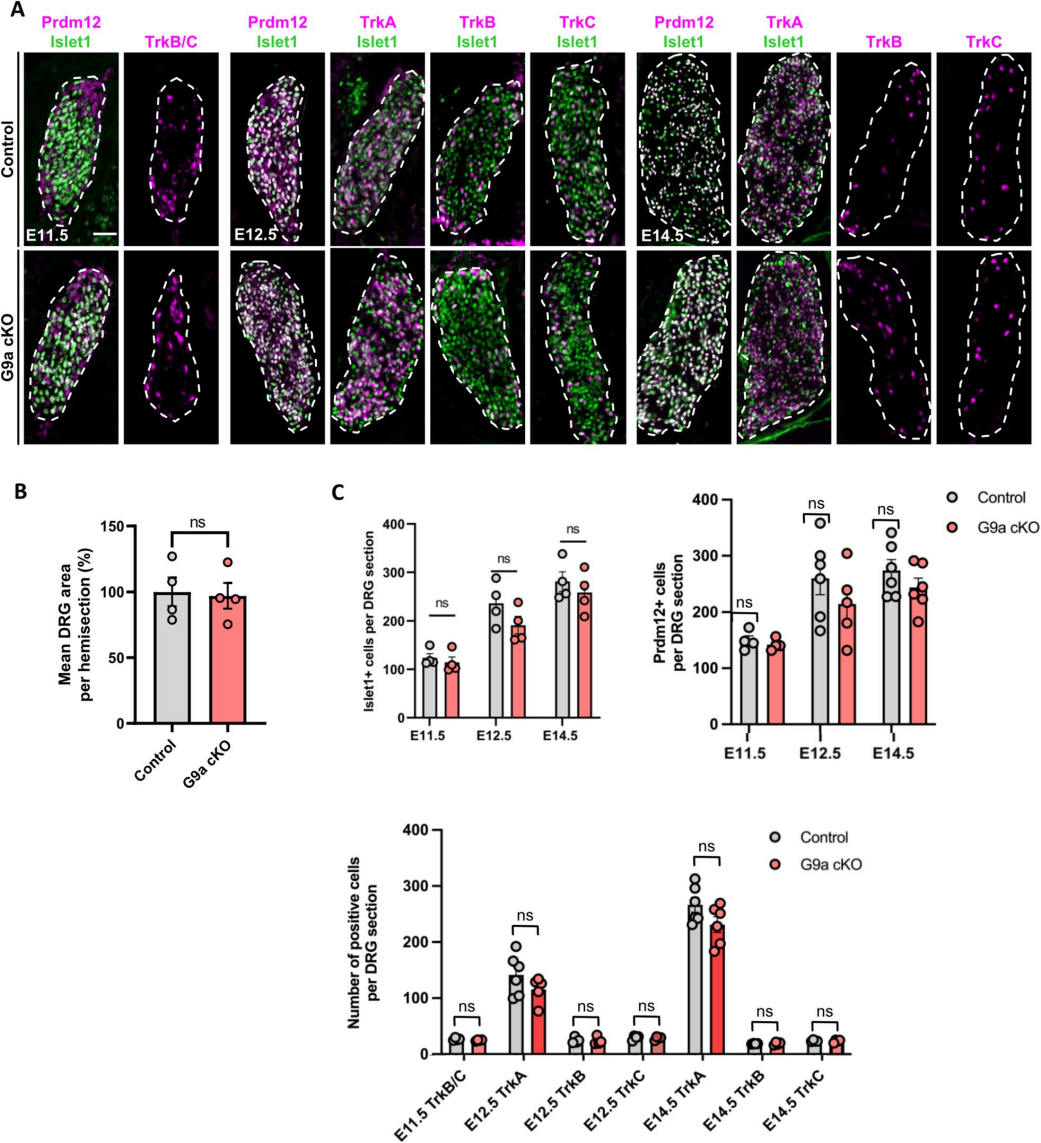
Loss of G9a is dispensable for early sensory neuron development in dorsal root ganglia. **A** Double immunostainings with indicated markers on coronal sections through DRG of control or *G9a* cKO embryos at E11.5, E12.5 and E14.5. Scale bars, 50 μm. DRG are delineated by white dashed lines. **B **Quantification of embryonic DRG area comparing E14.5 control and *G9a* cKO embryos. Histograms are represented as mean ± SEM.​ Each dot represents the mean value obtained for an individual biological replicate. Mann-Whitney test. *P*-value, ns > 0.999.​ **C** Quantification of the mean number of cells positive for the indicated markers on DRG coronal sections of control or *G9a* cKO embryos at the indicated stages. Histograms are represented as mean ± SEM. Each dot represents the mean value obtained for an individual biological replicate. Mann-Whitney test. *P*-value, ns > 0.999

In developing DRG, *Prdm12* is selectively expressed in the TrkA lineage which accounts for the vast majority of DRG somatosensory neurons. Following *Prdm12* knockout this whole lineage fails to develop, concomitantly resulting in a dramatic hypoplasia of DRG [11, 12]. To assess the role of G9a in DRG neurogenesis, we performed immunofluorescence staining using the pan-sensory neuronal marker Islet1 on coronal sections through DRG of E11.5 to E14.5 control and *G9a* cKO embryos. No significant difference in the number of Islet1^+^ neurons in DRG nor of DRG area size (delimitated using Islet1^+^ signals) was found between *G9a* cKO and control embryos, at any of the stages examined. Despite a trend for reduction, no significant difference in the number of nociceptive neurons as visualized by TrkA or Prdm12 immunostaining was also observed between *G9a* cKO and control embryos at E11.5, E12.5 and E14.5 (Fig. 3). This contrasts with the severe neuronal loss and DRG hypoplasia observed in *Prdm12* KO embryos from E13.5 onward following the agenesis of the TrkA neuron lineage [11, 12]. As G9a appears more broadly expressed than Prdm12 in embryonic DRG, we next also examined the consequence of the loss of *G9a* on the non-nociceptive neuron lineages, following the expression of the neurotrophic receptors TrkB and TrkC, labeling mechano/proprioceptive neurons. No difference was again observed using these markers between *G9a* cKO and controls (Fig. 3).

In embryonic DRG of *Prdm12* KO embryo, increased apoptosis and reduced cell proliferation have been reported to contribute to the agenesis of the TrkA neurons lineage [11–13]. Therefore, and given the importance of G9a in the control of cell proliferation and survival [30], we also investigated the consequences of the loss of *G9a* on cell death using immunostaining with anti-activated caspase 3 antibodies and on cell proliferation using phospho-Histone H3 antibodies. While no cell proliferation defect was detected in *G9a* cKO, a transient increase of apoptosis was found at E11.5, which was not detectable anymore at E14.5 (Figure S3). Thus, in *G9a* cKO embryos, a transient increase of apoptosis appears to occur that however does not result in a dramatic loss of the nociceptive lineage, as observed in *Prdm12* KO [11].

Phox2b is a master regulator of visceral fates in the peripheral nervous system [4, 31, 32]. As we recently discovered that Prdm12 also promotes nociceptor fate by repressing Phox2 genes and thus preventing precursors from engaging into an alternate visceral neuronal differentiation program [33], we also analyzed Phox2b expression in DRG of *G9a* cKO embryos. While Phox2b positive cells were indeed observed in E11.5 *Prdm12* KO DRG, none could be detected in *G9a* cKO (Figure S4).

Together, these results indicate that G9a is not essential for somatosensory neurogenesis. They suggest that it does not act as a critical mediator for Prdm12 functions in the initiation of the nociceptive neuron lineage.

### G9a appears dispensable for the maturation of the three main subtypes of somatosensory neurons

**Fig. 4:**
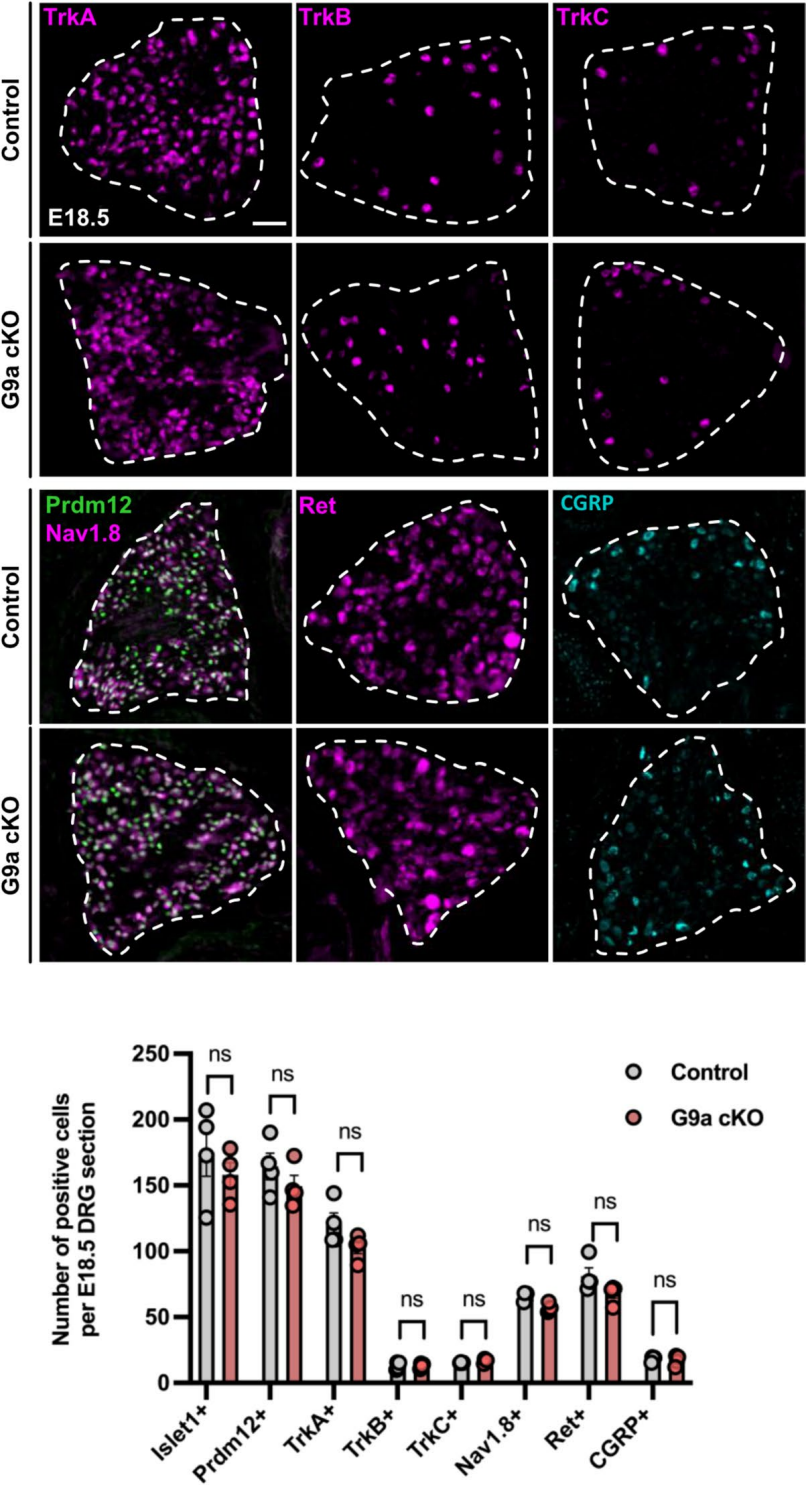
Loss of G9a is dispensable for the maturation of dorsal root ganglia sensory neurons. (Top panels) Immunostainings with indicated markers of sensory neuron subtypes performed on coronal sections through DRG of control or *G9a* cKO embryos at E18.5. Scale bars, 50 μm. DRG are delineated by white dashed lines. (Bottom panels) Quantification of the mean number of cells labelled with indicated markers on coronal sections through DRG of control or *G9a* cKO embryos at indicated stages. Histograms are represented as mean ± SEM. Each dot represents the mean value obtained for an individual biological replicate. Mann-Whitney test. *P*-value, ns > 0.999

Sensory neurogenesis in DRG begins around E9.5 and is complete by E14.5. It further overlaps and is followed by a phase of developmental maturation during which somatosensory neurons further refine into more specialized somatosensory subtypes [1, 3, 9]. To determine if G9a is involved in this sensory fate refinement phase, we examined the expression of sensory subtype markers at E18.5. At this stage, TrkB-expressing neurons label subtypes of myelinated LTMR neurons while TrkC is also found in LTMR subtypes and in proprioceptors [9]. The nociceptive component of the TrkA lineage also undergoes a functional refinement with the gradual emergence of peptidergic (PEP) nociceptors, which maintain TrkA and begin to express neuropeptides such as Calcitonin-Gene Related Peptide (CGRP), and non-peptidergic (NP) nociceptors which express the neurotrophic receptor Ret and initiate a phase of TrkA extinction [34]. Most neurons of the TrkA lineage also start to express the sodium channel Nav1.8 as a critical component driving their mature electrophysiological properties. We performed immunostainings using antibodies against all above cited markers as well as against Prdm12 and the pan-sensory neuron marker Islet1 to get clues of any maturational discrepancy of somatosensory subtypes in E18.5 control and *G9a* cKO DRG (Fig. 4). However, again, no significant difference was observed between *G9a* cKO and controls. Thus, G9a appears also dispensable for the maturation of the three main subtypes of somatosensory neurons.

## Discussion

PNS somatosensory neurons develop into specific subtypes thanks to the selective expression of a cascade of transcriptional regulators providing them with a discriminative transcriptional identity ultimately reflected by their subtype-specific functional diversity [3, 5]. At the root of the TrkA-lineage, from which emerge most if not all small diameter somatosensory neurons (i.e., nociceptors and C-LTMR), stands the histone methyltransferase (HMT) related transcriptional regulator Prdm12. In the recent years, several studies have shown how much Prdm12 is instrumental in the emergence of the TrkA-lineage, which fails to develop in its absence [10–13]. Mechanistically, Prdm12 appears to act as a pseudo methyltransferase as it has been shown to interact with G9a when overexpress in HEK29T3 cells and to increase H3K9me2 level when overexpressed in *Xenopus* neuralized animal cap explants [15, 22]. However, the in vivo relevance of G9a for Prdm12 functions in the emergence of the nociceptive lineage during somatosensory neurogenesis has never been investigated.

Here, using co-immunoprecipitation experiments, we confirm the ability of mPrdm12 to interact with G9a when overexpressed in HEK293T cells and that its first two ZF domains are required for this interaction. By contrast, Yildiz et al., 2019 found that the ZF domain of the zebrafish ortholog of Prdm12 is not required for this binding [35]. The origin of this discrepancy is unclear as the sequence of the two first zinc finger domains of the mouse and zebrafish Prdm12 proteins is identical. We further demonstrate that the SET domain of G9a is required and sufficient for the interaction with Prdm12. The C2H2 zinc finger proteins WIZ and ZNF644 have been also shown to interact with the SET domain of G9a [36, 37]. Like Prdm12, several other members of the Prdm family, including Prdm1, Prdm4, Prdm5, Prdm6 and Prdm16 have been shown to function as indirect epigenetic regulators and to be able to recruit G9a [38–41]. Whether like Prdm12 their association with G9a occurs with its SET domain remains to be determined. Using GST pull-down experiments, we provide evidence that Prdm12 can interact physically with G9a. Whether direct Prdm12-G9a interaction occurs in vivo in DRG remains however unclear. Indeed, G9a was not recovered in immunoprecipitation experiments performed using extracts prepared from dissected E12.5 embryonic DRG and Prdm12 specific antibodies followed by the identification by mass spectrometry of the co-immunoprecipitated proteins as well as in Rapid Immunoprecipitation Mass spectrometry of Endogenous proteins (RIME) experiments performed with V5 antibodies on fixed chromatin extracts prepared from dissected DRG of adult transgenic mice expressing a V5-tagged version of Prdm12 (unpublished data).

We show that during early somatosensory neurogenesis, G9a is broadly expressed in neural precursors and differentiating neurons, suggesting that G9a may play a role in Prdm12’s function in somatosensory neurogenesis. However, in G9a mutants, no deficiencies comparable to that of *Prdm12* KO embryos was observed. Specifically, in DRG of G9a cKO early embryos, neither vanishing of the TrkA-lineage, nor ectopic Phox2b expression was detected. At E18.5, no defect in the expression of late nociceptive markers was detected. We however observed at all stages examined a trend for a reduction in the number of Prdm12^+^ and TrkA^+^ neurons together with a transient increase of apoptosis at E11.5 in DRG of mutants. Although the identity of the additional dying cells remains unknown these observations together suggest that these additional dying cells may be developing nociceptors. The hypothesis that these additional dying cells are neuronal is also further supported by our observation that Sox10^+^ glial precursors appear also unaffected in DRG of G9a cKO embryos (Figure S5). Together, these data indicate that G9a is not essential for DRG neurogenesis. This contrasts with its important role in cranial neural crest for bone formation [42, 43] and in neurogenesis and neuronal maturation and function in other regions of the nervous system [44–46]. They suggest that Prdm12 function in developing DRG does not critically involves its interaction with G9a. In mature nociceptors, G9a plays an important role in neuropathic pain [47]. Whether Prdm12 later functions in the modulation of the excitability of mature nociceptors involves its interaction with G9a remains to be investigated.

Despite the fact that in the DRG of G9a cKO embryos, H3K9me2 level continues to be reduced during late neurogenesis (Fig. Sup 2C), whether the lack of phenotype is due to some redundancy or compensation mechanisms is a possibility that cannot however be excluded. The G9a related histone methyltransferase GLP that dimerizes with G9a to catalyze histone H3 lysine 9 mono- or di-methylation is one candidate that may compensate G9a. This is however uncertain as we found that, as previously reported in early embryos [48], the level of H3K9me2 is already drastically reduced in DRG in the absence of G9a alone (Fig. S2C) and G9a depletion is known to destabilize GLP in cranial neural crest [42]. Besides, a compensation of G9a by GLP for Prdm12 function in nociceptor development appears unlikely as GLP does not appear to bind to Prdm12 [22].

In the Biogrid protein-protein interaction database, the histone methyltransferase Enhancer of zeste homolog 2 (EZH2), the catalytic component of the polycomb repressive complex 2 (PCR2) that adds methyl groups to lysine 27 of histone H3 (H3K27), and thereby represses target genes [49] has been identified as another putative Prdm12 interacting partner. Besides, EZH2 and G9a have been shown to functionally cooperate in gene silencing in ES cells [50] and in the induction of tumor cell death [51]. We thus evaluated EZH2 expression by immunostaining and found that it is largely coexpressed with Prdm12 in DRG of developing mouse embryos (Fig. S6A). We also validated experimentally the ability of Prdm12 to interact with EZH2 when overexpressed in HEK293T cells using Co-IP analysis and obtained evidence that the domain within Prdm12 and EZH2 that are required for the interaction are distinct to those mediating Prdm12-G9a interaction (Fig. S6B). Whether EZH2 compensates G9a loss in DRG is here again uncertain as EZH2 loss in trunk neural crest has been shown not to interfere with neuronal differentiation [52]. Studies using double knock-out for these two interactors are needed to determine whether they cooperate in the control of DRG development.

In conclusion, our data indicate that Prdm12 interaction with G9a is not obligatory for its function in the initiation of the nociceptive lineage. They suggest the existence of other Prdm12 interacting partners that may play a more instrumental role in its functional properties. Whether in the initiation of the nociceptive lineage, Prdm12 necessarily involves a methyltransferase activity remains thus today unclear. Defining Prdm12’s interactome in DRG in an unbiased manner will thus be of paramount importance to understand its mechanism of action.

## Material and methods

### Mouse ethics and crossing strategy

All mice were maintained on a C57BL/6J background. Mice were housed at room temperature with a 12h light/dark cycle in standard cages with litter, water and food *ad libitum*. Air circulation in the facility was filtered and temperature monitored at a steady 20^o^C. Cages were also provided with cottons and cardboard rolls for enrichment. The experimental protocols were approved by the CEBEA (Comité d’éthique et du bien-être animal) of the IBMM-ULB and conform to the European guidelines on the ethical care and use of animals. The following mice strains were used: *Prdm12*^*LacZ/LacZ*^ [11], *Ehmt2/G9a*^*fl/fl*^ (generated in Y. Shinkai’s laboratory [28] and kindly provided by Prof. Maite García Fernández de Barrena, Universidad de Navarra) and *Wnt1*^*Cre*^ [53].

For the generation of the *Wnt1*^*Cre*^; *G9a*^*fl/fl*^mouse line (G9a cKO), *Wnt1*^*Cre*^males and females were crossed with *G9a*^*fl/+*^or *G9a*^*fl/fl*^females and males, respectively. *Wnt1*^*Cre*^; *G9a*^*fl/+*^mice were then crossed with *G9a*^*fl/fl*^mice to maintain the line and obtain control (*G9a*^*fl/+*^, *G9a*^*fl/fl*^or *Wnt1*^*Cre*^; *G9a*^*fl/+*^) and *G9a* cKO embryos. For embryo harvesting, the day of vaginal plug was considered to be embryonic day (E) 0.5. A minimum of 8 sections per tissue and at least 4 embryos of the same genotype were analyzed in each experiment. Embryos were collected at E10.5, E11.5, E12.5, E14.5 and E18.5.

Polymerase Chain Reaction (PCR) was used for genotyping of the collected embryos as follows: For the *G9a* floxed and WT alleles, using primers forward 5’-CTGCACGCTGCCTAGATGGAGCATG-3’ and reverse 5’- CTGGGTGGAAAGTTGCCAGGCTTAG-3’, for the *Wnt1*^*Cre*^transgene, using primers forward 5′-CCACCTCTTCGGCAAGATCG-3′ and reverse 5′-GCTAGAAAGAATCTGGTGCTGACC-3′, for the *Prdm12*^*LacZ*^andWT alleles, using primers forward 5’-AGTTTGTACATTCCCTGGGAGTAAGACTCC-3’ and reverse 5’-AGCCAGGGGAAGAATGTGAGTTGC-3’.

### Plasmids and cloning

Flag-mPrdm12 WT and mutant and G9a (S) expression plasmids were kindly provided by Prof. Yoichi Shinkai, University of Kyoto, Japan [22]. G9a deletion mutants were generated by PCR amplification or overlap extension PCR and inserted in frame with the MYC tag into the pCS2-MT-NLS expressing vector using the In Fusion Protocol (ST0345, Takara). All constructs were confirmed by Sanger sequencing.

### Cell cultures and Co-immunoprecipitation assays

Human embryonic kidney cells (HEK293T) were maintained in T-75 culture flasks at 37^o^C and 5% CO_2_. DMEM medium (Gibco) was supplemented with 10% fetal bovine serum (FBS, Gibco), 100 U/ml penicillin/streptomycin (Gibco) and 1mM sodium pyruvate (Gibco) (Maintenance Medium). Cells were subcultured when reaching 80 – 90% confluency. All media was replaced every 48 hours.

For transfection assays, HEK293T cells were plated on coated 10cm culture dishes (Greiner Bio-One, vented, sterile, PS coating) at a confluency of 3 x 10^6^cells/dish. Dishes were used for plasmid transfection after reaching 50-80% confluency, usually 24h-48h after plating. 18-20 μg of indicated plasmids were transfected in HEK293T cells using the CalPhos Mammalian Transfection Kit (Takara). After 48h, cells were washed with RNAse Free ice-cold PBS and lysed for 15 - 20 mins in IPH Lysis Buffer (150 mM NaCl, 5 mM EDTA pH 8.0, 50 mM Tris pH8.0, 1% NP-40) containing protease inhibitor cocktail (cOmplete, EDTA-free Protease Inhibitor Cocktail – Roche). Supernatants were collected after centrifugation at 12000 RPM, 4^o^C and precleared by incubating with a mix of Protein G Plus/A Agarose Beads (Millipore) on a tube rotator for 2-3h, 4^o^C. Protein concentration was estimated with DC Protein Assay (Biorad) and equal amounts of protein (25 μg) were mixed with 5x Laemmli Buffer, heated up for 5 min, 95^o^C and run on 10% SDS-PAGE gels and transferred to nitrocellulose membranes (Amersham Protran Western blotting membranes – Sigma Aldrich) to validate protein quality and specificity. 48h after transfection, immunoprecipitations were performed with 1000 μg of total protein extracts from transfected cells and 5 μg of antibody overnight at 4°C under rotation. The day after, 40 μl of Protein A Sepharose CL-4B beads (Sigma; GE17-0780-01) was added in the tube and incubated for and additiona1 hour at 4°C under rotation. After three washes with IPH buffer 150 mM, immunoprecipitated proteins were eluted by heating at 100°C during 5 minutes in 1 x Laemmli Sample Buffer and subjected to Western blot analysis.

### Quantification of embryonic DRG area

Fourteen µm serial sections of the thoracic part of E14.5 embryos were collected starting at the heart level and finishing before reaching the level of the liver with intervals of 112 µm. Sections were immunostained for the pan-neuronal marker Islet1 to allow the proper delimitation of the whole DRG area per hemisection. Pictures of consecutive hemisections (5 on the left and 5 on the right) were taken to determine the mean DRG area per hemisection for each biological replicate. If a DRG was visible on the hemisection, Islet1+ signal was used to delineate and calculate the area of the DRG using the image analysis software Fiji/ImageJ. If no DRG was visible on a specific hemisection, the DRG area considered to be 0 for this specific hemisection. DRG area were then reported as percentages with the mean value of control replicates being thresholded as 100%.

### Immunofluorescence

Dissected embryos were fixed in 4% Paraformaldehyde (PFA) for 15 minutes, washes 4 times with ice cold phosphate-buffered saline (PBS) and then, cryoprotected at 4°C overnight in 30% Sucrose dissolved in PBS. Embryos were embedded in 7,5% gelatin – 15% sucrose (dissolved in PBS) and stored at -80oC. The blocks are sectioned at the level of the thoracic dorsal root ganglia into 14μm sections at -30oC in the cryostat and collected slides are kept at -20oC. Immunostainings were performed as previously described [23] using mouse monoclonal α-H3K9me2 (Abcam, ab1220),  rabbit polyclonal α-G9a (Abcam Ab229455), mouse monoclonal anti-SOX10 (Abcam, ab216020), Goat polyclonal α-TrkA (R&D Systems, AF1056), Goat polyclonal α-TrkB (Cell Signaling, AF1494), Goat polyclonal α-TrkC (Cell Signaling, AF1404), Chicken polyclonal α-Peripherin (Abcam, ab106276), Rabbit polyclonal α-pH3 (Millipore, 07-690), Rabbit polyclonal cleaved α-Caspase (Cell Signaling, 9661), Rabbit polyclonal α-Phox2b (kind gift from Jean-François Brunet), Rabbit polyclonal α-Nav1.8 (Abcam, ab63331), Goat polyclonal α-Ret (R&D Systems, AF482), Rabbit polyclonal α-CGRP (Sigma-Aldrich, C8198), Mouse monoclonal α-Flag (Sigma-Aldrich, F1804), Rabbit polyclonal α-c-Myc (Sigma-Aldrich, PLA0001) and Normal Rabbit IgG (Cell Signaling, 2729). Secondary antibodies used in this study were Goat α-mouse IgG HRP (Jackson ImmunoResearch, 115-035-003), Goat α-rabbit IgG HRP (Cell Signaling, 7074), Goat α-mouse Alexa 594 (Invitrogen, A11032), Goat- α-rabbit Alexa Fluor 488 (Invitrogen, A11008), Goat α-rabbit Alexa Fluor 594 (Invitrogen, A11012), Goat α-guinea pig Alexa Fluor 488 (Invitrogen, A11073), Goat α-guinea pig Alexa Fluor 594 (Invitrogen, A11076), donkey α-mouse 488 (Invitrogen, A21202), donkey α-goat Alexa Fluor 594 (Invitrogen, A11058. Immunofluorescent images were acquired on a Zeiss Axio Observer Z1 fluorescent microscope or a laser-scanning confocal microscope Zeiss LSM 710 using the Zeiss Zen 2 microscope software. Analysis of the fluorescence intensity of H3K9me2 in E14.5 DRG sections was performed using FIJI/ImageJ. Briefly, five DRG sections per individual were taken under a LSM 710 confocal microscope with the exact same parameters of acquisition. Control and mutants embryos sections were collected two by two on the same slides and all replicates were immunostained at the same time and imaged on the same day to reduce the variability of the immunostaining and signal acquisition. The fluorescence level reported for each individual corresponds to the mean of fIuorescence level calculated with the "Measure” tool of FIJI/ImageJ for each five DRG sections. In each picture, the fluorescence level was determined by measuring the average fluorescence intensity of H3K9me2 in DRG sections delineated via DAPI counterstain.

### RT – qPCR

Total RNA from dissected E14.5 DRG was extracted using the Monarch Total RNA Miniprep Kit (New England Biolabs). cDNA was synthesized with iScript cDNA synthesis kit (Biorad) and RT-qPCR was performed using the Luna Universal qPCR Master Mix (New England Biolabs). The comparative 2^-^^ΔΔ^^CT^method was used to determine relative expression of the *G9a* cKO samples to compared the expression level of controls, overall normalized to *GAPDH* expression. The following primers were used: For *GAPDH*, forward primer 5’- CTCCCACTCTTCCACCTTCG -3’ and reverse 5’- GCCTCTCTTGCTCAGTGTCC -3’, for *G9a/Ehmt2 *forward primer 5’- CTCTACCGGACTGCCAAGAT -3’ and reverse 5’- CTCGGCATCAGAGATCAGC -3’ (Ideno et al, 2020). Two-tailed Student’s t-test was used to measure statistical significance (* = *p* value <0.05, sample pool size *n*=4).

### Statistical analysis

Statistical analyses were performed using GraphPad version 9. Cell countings were performed for at least 4 biological replicates per condition, on at least 8 DRG sections per sample. For statistical analysis, the unpaired, non-parametric Mann Whitney test was used (*=*p*value <0.05). Graphical quantifications were represented from qualitative data indicating the number (n) of embryos included in the analysis as individual values (mean +/- SEM).

### Supplementary Information

**Additional file 1:** **Figure S1.** G9a and Sox10 expression are mainly non-overlapping in DRG of E15.5 embryos. Immunostainings for G9a and Sox10 are shown on coronal sections through DRG of wild-type mouse embryos at E15.5. Quantification of the mean number of Sox10+ expressing cells is shown on the right. Histograms are represented as mean ± SEM (*n* = 2). Scale bars, 50 µm. **Figure S2.** Validation of the G9a conditional knockout (cKO) mouse model. (A) Double immunostaining with the pan-sensory neuron marker Islet1 and G9a antibodies performed on coronal sections through DRG of control or *G9a *cKO embryos at E12.5. Scale bar, 50 µm. Quantification of the mean number of G9a-positive neurons on DRG coronal sections of control or *G9a *cKO embryos at E11.5 and E12.5 is shown on the right. Each dot represents the mean value of G9a+ neurons in one biological replicate. Mann-Whitney test. *P*-value, * < 0.005. Mean ± SEM (B) Relative expression of *G9a *quantified by RT-qPCR in DRG collected from E14.5 control and *G9a *cKO embryos. Mean ± SD,*n*=3. Student’s T- test with Welch correction. *P*-value, * <0.005. (C) Left panels: double immunostaining with antibodies against Six1 and the histone methylation mark H3K9me2 on coronal sections through DRG of control, *G9a *cKO or *Prdm12 *KO embryos at E11.5. Scale bar, 50 µm. DRG are delineated by white dashed lines. Right panels: immunostaining against H3K9me2 on coronal sections through DRG of control and *G9a *cKO embryos counterstained with DAPI at E14.5. Scale bar, 50 µm. Bottom: quantification of the H3K9me2 immunostaining mean fluorescence level (arbitrary unit, A.U.) detected on DRG sections of control and *G9a *cKO embryos at E14.5. Mann-Whitney test. *P*-value, * < 0.005. Mean ± SEM. **Figure S3.** Loss of G9a results in a transient increase of apoptosis in DRG. (A) Double immunostainings with antibodies against the pan-neuronal marker Islet1 and the pro-apoptotic Cleaved- Caspase3 (upper panels) or against phospho-Histone H3 (PH3, lower panels) on coronal sections through DRG of control or *G9a*cKO embryos at E11.5 and E14.5. Scale bar, 50 µm. DRG are delineated by white dashed lines. (B) Quantification of the mean number of Caspase3+ cells on coronal sections through DRG of control or *G9a *cKO embryos at indicated embryonic stages. (C) Quantification of the mean number of PH3+ cells on coronal sections through DRG of control or *G9a *cKO embryos at indicated embryonic stages. Histograms are represented as mean ± SEM. Each dot represents the mean value obtained for an individual biological replicate. Mann-Whitney test. *P*-value, * <0.005, ns > 0.999. **Figure S4.** Loss of G9a does not induce Phox2b ectopic expression as observed upon loss of Prdm12. Double immunostainings with antibodies against the pan-neuronal marker Islet1 and the transcription factor Phox2b on coronal sections through DRG of control, *Prdm12 *KO and *G9a *cKO embryos at E11.5. Scale bar, 50 µm. DRG are delineated by white dashed lines. **Figure S5.** Loss of G9a does not affect the number of Sox10+ glial precursors in DRG of E14.5 embryos. (A) Immunostainings with Sox10 and Prdm12 antibodies on coronal sections through DRG of control and *G9a *cKO embryos. Scale bar, 50 µm. (B) Quantification of the relative proportion of Sox10+ glial cells calculated as the ratio of Sox10+ cells/Prdm12+ cells per DRG hemisection, subsequently expressed as percentage. Histograms are represented as mean ± SEM. Each dot represents the mean value obtained for an individual biological replicate. Mann-Whitney test. *P*-value, ns > 0.999. **Figure S6.** Prdm12 interacts with EZH2 and the first two zinc fingers of Prdm12 and the SET domain of EZH2 are not required for this interaction. (A) High magnification views of immunostainings for Prdm12 and EZH2 on coronal sections through DRG of wild-type mouse embryos at indicated stages. Quantification of the mean number of Prdm12+ cells or Prdm12+/EZH2+ cells detected in coronal sections through DRG of wild-type embryos at indicated stages is shown on the right. Histograms are represented as mean ± SEM. Each dot represents the mean value obtained for an individual biological replicate. Scale bars, 50 µm. (B) HEK293T cells were transfected with the indicated plasmids. Schematic diagram of WT and deletion mutants of Flag-PRDM12 and Myc-EZH2 are shown. An empty FLAG vector was used as a control. Lysates were immunoprecipitated with anti-Flag antibodies. Immunoprecipitates and 5% of the input were then subjected to western blot analysis with anti-Flag or anti-Myc antibodies.

## Data Availability

All data used in the preparation of this manuscript will be provided upon request.
