# Supplementary material for: Loss of G9a does not phenocopy the requirement for Prdm12 in the development of the nociceptive neuron lineage
Source: Neural Dev. 2024 Jan 2;19:1. doi: 10.1186/s13064-023-00179-7 (PMC10759634; doi:10.1186/s13064-023-00179-7)
Supplement: Supplementary file 1 — Additional file 1: Figure S1. G9a and Sox10 expression are mainly non-overlapping in DRG of E15.5 embryos. Immunostainings for G9a and Sox10 are shown on coronal sections through DRG of wild-type mouse embryos at E15.5. Quantification of the mean number of Sox10+ expressing cells is shown on the right. Histograms are represented as mean ± SEM (n = 2). Scale bars, 50 µm. Figure S2. Validation of the G9a conditional knockout (cKO) mouse model. (A) Double immunostaining with the pan-sensory neuron marker Islet1 and G9a antibodies performed on coronal sections through DRG of control or G9a cKO embryos at E12.5. Scale bar, 50 µm. Quantification of the mean number of G9a-positive neurons on DRG coronal sections of control or G9a cKO embryos at E11.5 and E12.5 is shown on the right. Each dot represents the mean value of G9a+ neurons in one biological replicate. Mann-Whitney test. P-value, * < 0.005. Mean ± SEM (B) Relative expression of G9a quantified by RT-qPCR in DRG collected from E14.5 control and G9a cKO embryos. Mean ± SD,n=3. Student’s T- test with Welch correction. P-value, * <0.005. (C) Left panels: double immunostaining with antibodies against Six1 and the histone methylation mark H3K9me2 on coronal sections through DRG of control, G9a cKO or Prdm12 KO embryos at E11.5. Scale bar, 50 µm. DRG are delineated by white dashed lines. Right panels: immunostaining against H3K9me2 on coronal sections through DRG of control and G9a cKO embryos counterstained with DAPI at E14.5. Scale bar, 50 µm. Bottom: quantification of the H3K9me2 immunostaining mean fluorescence level (arbitrary unit, A.U.) detected on DRG sections of control and G9a cKO embryos at E14.5. Mann-Whitney test. P-value, * < 0.005. Mean ± SEM. Figure S3. Loss of G9a results in a transient increase of apoptosis in DRG. (A) Double immunostainings with antibodies against the pan-neuronal marker Islet1 and the pro-apoptotic Cleaved- Caspase3 (upper panels) or against phospho-Histone H3 (PH3, lower panels) on co [file 13064_2023_179_MOESM1_ESM.pdf]

Supplementary Figure 1

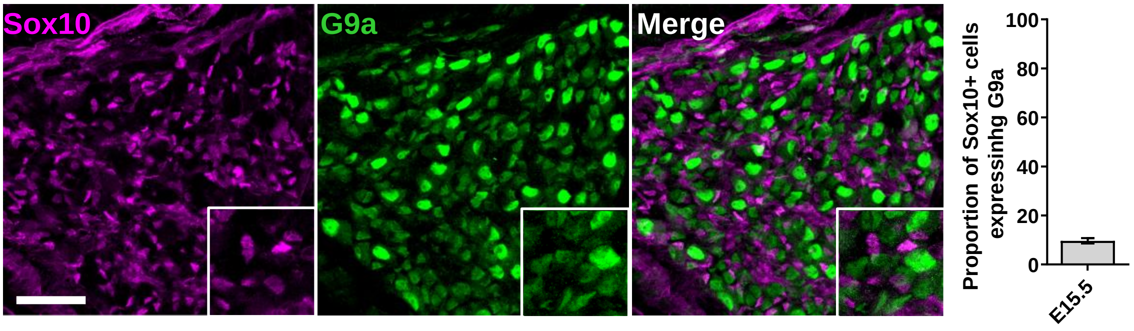

Figure S1. G9a and Sox10 expression are mainly non-overlapping in DRG of E15.5 embryos. Immunostainings for G9a and Sox10 are shown on coronal sections through DRG of wild-type mouse embryos at E15.5. Quantification of the mean number of Sox10^{+} expressing cells is shown on the right. Histograms are represented as mean ± SEM (n = 2). Scale bars, 50 µm.

Supplementary Figure 2

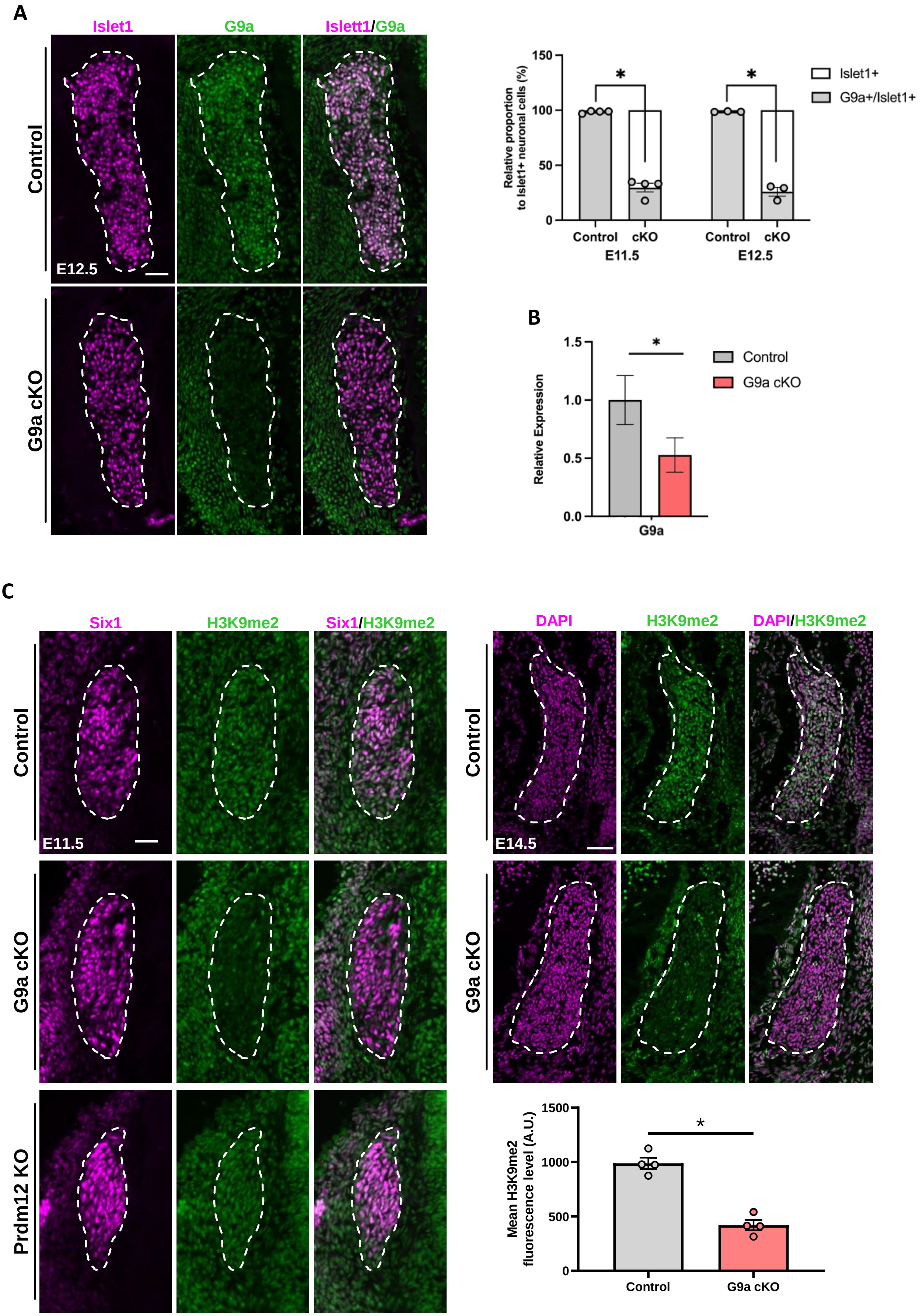

Figure S2. Validation of the G9a conditional knockout (cKO) mouse model. (A) Double immunostaining with the pan-sensory neuron marker Islet1 and G9a antibodies performed on coronal sections through DRG of control or *G9a* cKO embryos at E12.5. Scale bar, 50 µm. Quantification of the mean number of G9a-positive neurons on DRG coronal sections of control or *G9a* cKO embryos at E11.5 and E12.5 is shown on the right. Each dot represents the mean value of $G9a^{+}$ neurons in one biological replicate. Mann-Whitney test. P-value, * < 0.005. Mean ± SEM **(B)** Relative expression of *G9a* quantified by RT-qPCR in DRG collected from E14.5 control and *G9a* cKO embryos. Mean ± SD, n=3. Student's T-test with Welch correction. P-value, * <0.005. **(C)** Left panels: double immunostaining with antibodies against Six1 and the histone methylation mark H3K9me2 on coronal sections through DRG of control, *G9a* cKO or *Prdm12* KO embryos at E11.5. Scale bar, 50 µm. DRG are delineated by white dashed lines. Right panels: immunostaining against H3K9me2 on coronal sections through DRG of control and *G9a* cKO embryos counterstained with DAPI at E14.5. Scale bar, 50 µm. Bottom: quantification of the H3K9me2 immunostaining mean fluorescence level (arbitrary unit, A.U.) detected on DRG sections of control and *G9a* cKO embryos at E14.5. Mann-Whitney test. P-value, * < 0.005. Mean ± SEM.

Supplementary Figure 3

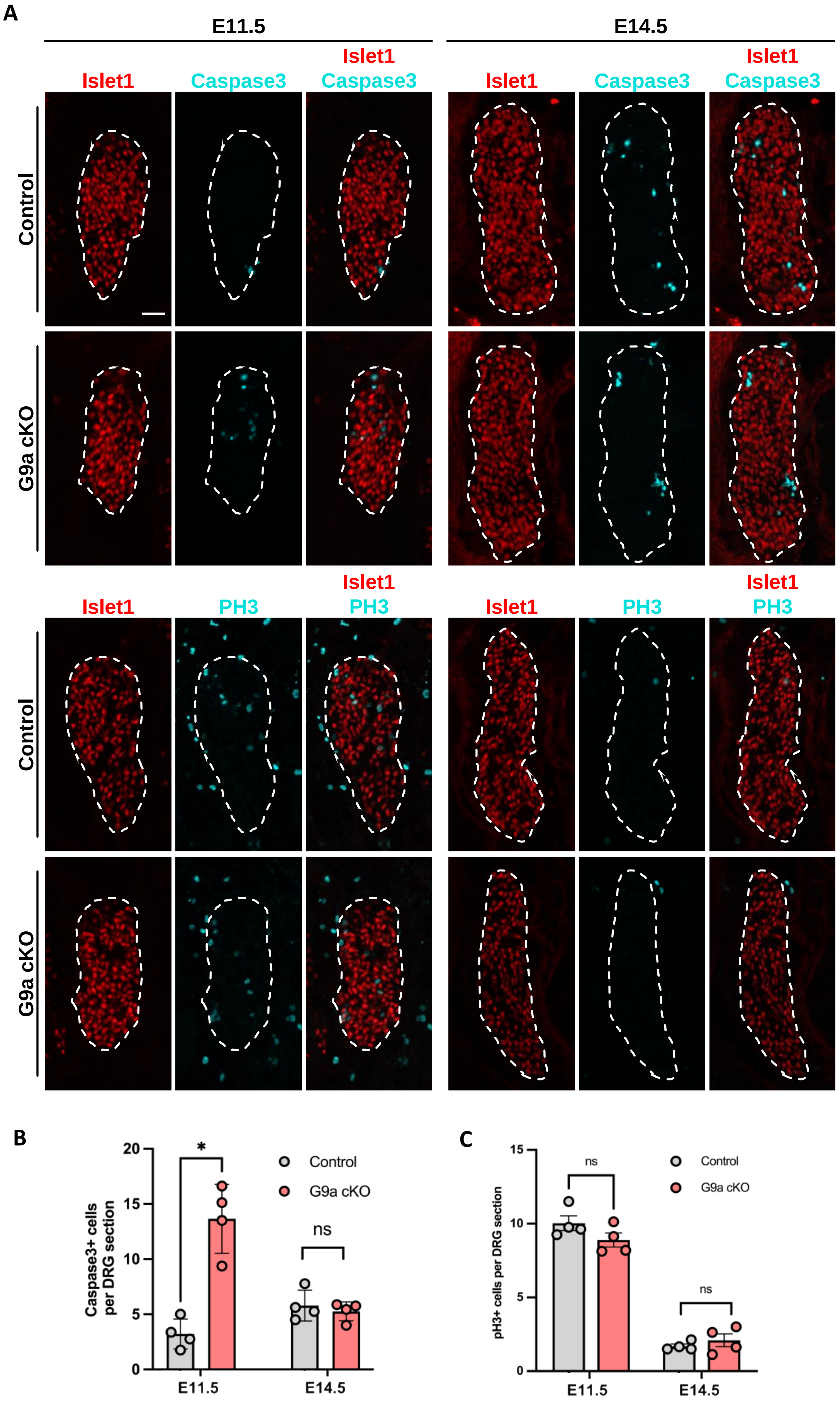

Figure S3. Loss of G9a results in a transient increase of apoptosis in DRG. (A) Double immunostainings with antibodies against the pan-neuronal marker Islet1 and the pro-apoptotic Cleaved-Caspase3 (upper panels) or against phospho-Histone H3 (PH3, lower panels) on coronal sections through DRG of control or *G9a* cKO embryos at E11.5 and E14.5. Scale bar, 50 µm. DRG are delineated by white dashed lines. **(B)** Quantification of the mean number of Caspase3$^+$ cells on coronal sections through DRG of control or *G9a* cKO embryos at indicated embryonic stages. **(C)** Quantification of the mean number of PH3$^+$ cells on coronal sections through DRG of control or *G9a* cKO embryos at indicated embryonic stages. Histograms are represented as mean ± SEM. Each dot represents the mean value obtained for an individual biological replicate. Mann-Whitney test. P-value, * <0.005, ns > 0.999.

Supplementary Figure 4

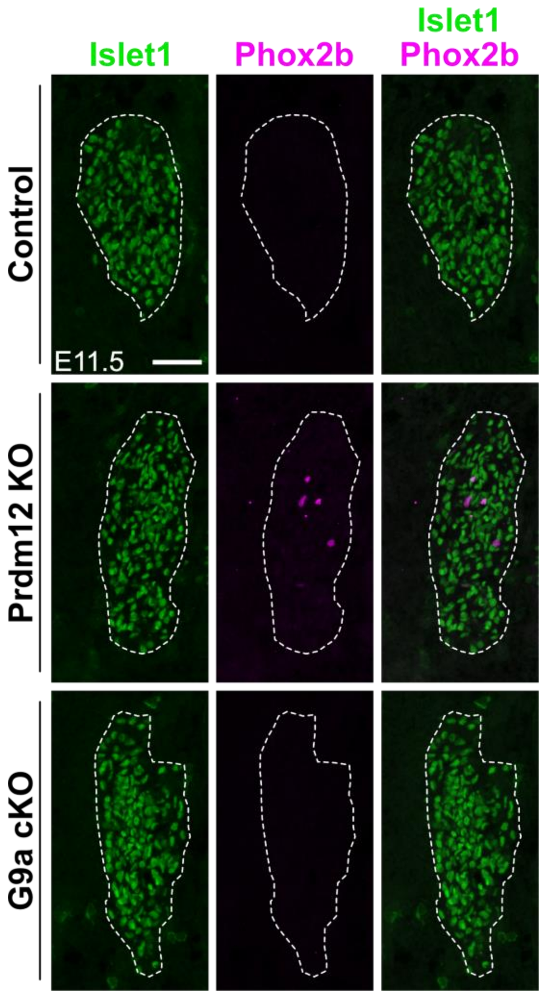

Figure S4. Loss of G9a does not induce Phox2b ectopic expression as observed upon loss of Prdm12. Double immunostainings with antibodies against the pan-neuronal marker Islet1 and the transcription factor Phox2b on coronal sections through DRG of control, *Prdm12* KO and *G9a* cKO embryos at E11.5. Scale bar, 50 µm. DRG are delineated by white dashed lines.

Supplementary Figure 5

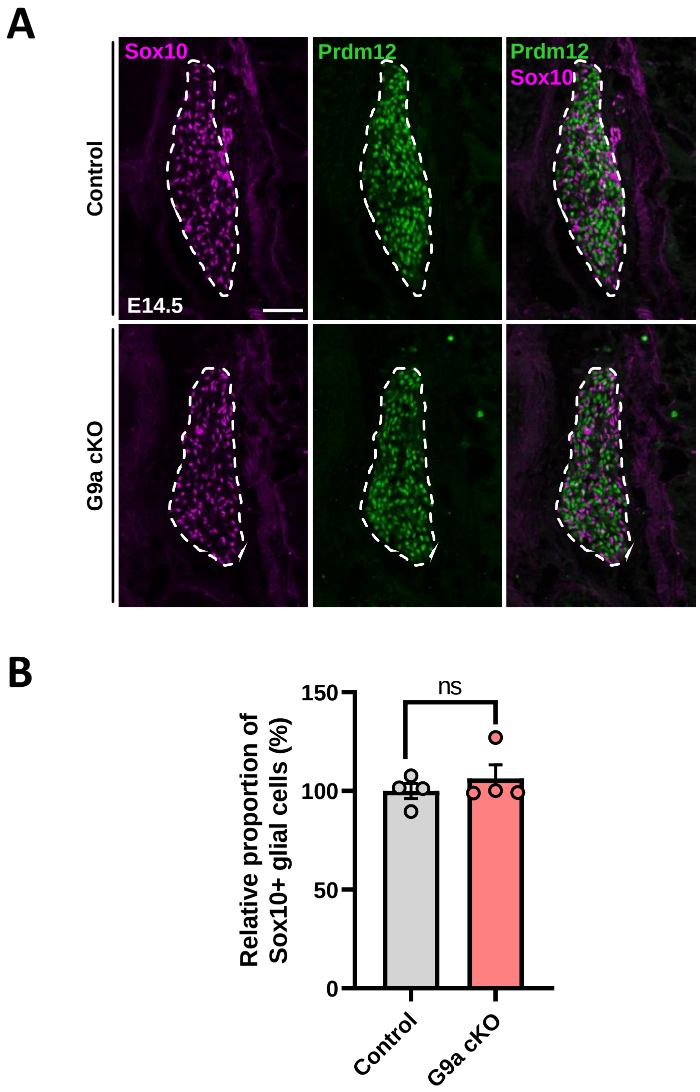

Figure S5. Loss of G9a does not affect the number of Sox10^{+} glial precursors in DRG of E14.5 embryos. (A) Immunostainings with Sox10 and Prdm12 antibodies on coronal sections through DRG of control and *G9a* cKO embryos. Scale bar, 50 µm. (**B**) Quantification of the relative proportion of Sox10^{+} glial cells calculated as the ratio of Sox10^{+} cells/Prdm12^{+} cells per DRG hemisection, subsequently expressed as percentage. Histograms are represented as mean ± SEM. Each dot represents the mean value obtained for an individual biological replicate. Mann-Whitney test. P-value, ns > 0.999.

Supplementary Figure 6

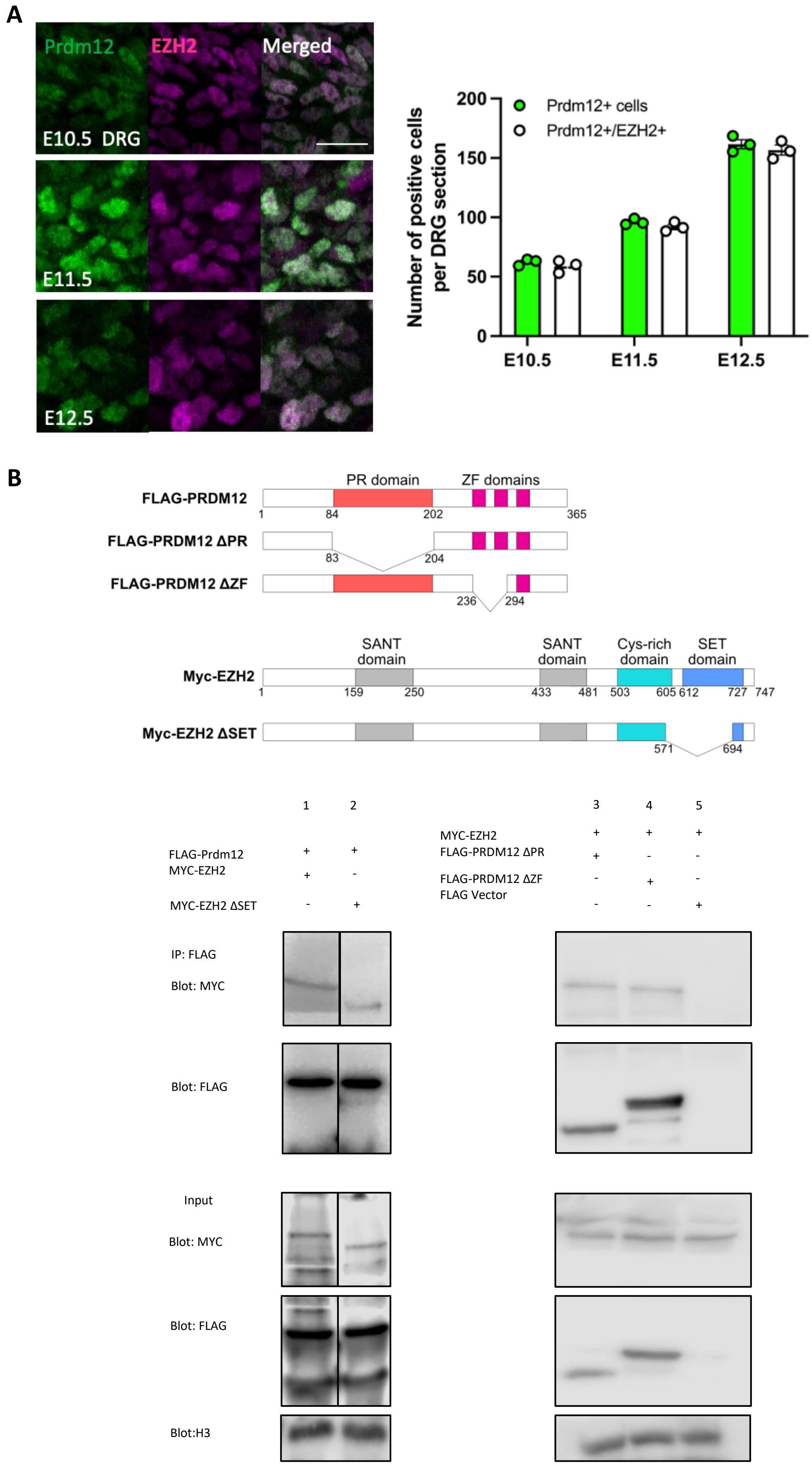

Figure S6. Prdm12 interacts with EZH2 and the first two zinc fingers of Prdm12 and the SET domain of EZH2 are not required for this interaction. (A) High magnification views of immunostainings for Prdm12 and EZH2 on coronal sections through DRG of wild-type mouse embryos at indicated stages. Quantification of the mean number of $Prdm12^{+}$ cells or $Prdm12^{+}/EZH2^{+}$ cells detected in coronal sections through DRG of wild-type embryos at indicated stages is shown on the right. Histograms are represented as mean ± SEM. Each dot represents the mean value obtained for an individual biological replicate. Scale bars, 50 µm. **(B)** HEK293T cells were transfected with the indicated plasmids. Schematic diagram of WT and deletion mutants of Flag-PRDM12 and Myc-EZH2 are shown. An empty FLAG vector was used as a control. Lysates were immunoprecipitated with anti-Flag antibodies. Immunoprecipitates and 5% of the input were then subjected to western blot analysis with anti-Flag or anti-Myc antibodies.
